# Assessing COVID-19-related health literacy and associated factors among school teachers in Hong Kong, China

**DOI:** 10.3389/fpubh.2022.1057782

**Published:** 2022-12-07

**Authors:** Sam S. S. Lau, Eric N. Y. Shum, Jackie O. T. Man, Ethan T. H. Cheung, Padmore Adusei Amoah, Angela Y. M. Leung, Kevin Dadaczynski, Orkan Okan

**Affiliations:** ^1^Research Centre for Environment and Human Health, School of Continuing Education, Hong Kong Baptist University, Kowloon, Hong Kong SAR, China; ^2^College of International Education, Hong Kong Baptist University, Kowloon, Hong Kong SAR, China; ^3^Multidisciplinary Research Centre, School of Continuing Education, Hong Kong Baptist University, Kowloon, Hong Kong SAR, China; ^4^Institute of Bioresource and Agriculture, Hong Kong Baptist University, Kowloon, Hong Kong SAR, China; ^5^Department of Applied Psychology, School of Graduate Studies, Institute of Policy Studies, Lingnan University, Tuen Mun, China; ^6^School of Nursing, Hong Kong Polytechnic University, Kowloon, Hong Kong SAR, China; ^7^Public Health Centre Fulda, Fulda University of Applied Sciences, Fulda, Germany; ^8^Center for Applied Health Science, Leuphana University Lueneburg, Lueneburg, Germany; ^9^Department of Sport and Health Sciences, Technical University Munich, Munich, Germany

**Keywords:** COVID-19, teachers, school, Hong Kong, China, vaccine hesitancy, corona-related health literacy

## Abstract

**Objectives:**

The coronavirus disease 2019 (COVID-19) pandemic developed rapidly, with changing guidelines, misinformation, inaccurate health information and rumors. This situation has highlighted the importance of health literacy, especially among educators. The aims of this study were (i) to assess COVID-19-specific health literacy among school teachers in Hong Kong and (ii) to examine its association with demographic factors, self-endangering work behaviors (i.e., work intensification, work extensification and work quality reduction), secondary burnout symptoms (i.e., exhaustion related to work and psychosomatic complaints), the level of knowledge of COVID-19- or pandemic-related information and the level of confusion about COVID-19-related information.

**Methods:**

A self-report survey was administered to 366 Hong Kong school teachers from April 2021 to February 2022. COVID-19-specific health literacy was measured using the HLS-COVID-Q22 instrument. Other instruments, including self-endangering work behavior scales (i.e., extensification of work, intensification of work and work quality reduction) and two dimensions of the Burnout Assessment Tool (i.e., psychosomatic complaints and exhaustion) were also used for assessment. Data were analyzed using an independent samples Student's *t-*test, analysis of variance, correlation analysis and adjusted multilinear regression models.

**Results:**

The results showed that 50.8% of school teachers had sufficient health literacy, 38.3% had problematic health literacy and 10.9% had inadequate health literacy. The HLS-COVID score did not vary by sex, but varied according to the type of school, the number of working hours per week and the number of students attending the school. Teachers with sufficient health literacy scored significantly lower for two types of self-endangering work behavior–intensification of work (*p* = 0.003) and work quality reduction (*p* = 0.007)—than those with insufficient health literacy. After excluding those who had already been vaccinated, respondents with sufficient health literacy felt more positive about COVID-19 vaccination than those with insufficient health literacy (t[180] = 4.168, *p* < 0.001). In addition, teachers with sufficient health literacy felt more informed (*p* < 0.001) and less confused (*p* < 0.001) about COVID-19-related information than those with insufficient health literacy. Multiple linear regression analysis revealed that age (β = 0.14, *p* = 0.011) and the number of teaching hours per week (β = −0.206, *p* < 0.001) were significant predictors of the HLS-COVID score.

**Conclusions:**

The findings of this study may serve as a guide for addressing health literacy gaps among school teachers.

## Introduction

The coronavirus disease 2019 (COVID-19) pandemic developed rapidly, with the continuous emergence of variants, such as the Omicron and Alpha variants (1). Such a rapid pandemic development increased the need for individuals to acquire and apply accurate health information at a fast pace (2). However, the COVID-19 pandemic has been accompanied by changing guidelines, misinformation, inaccurate health information and rumors, resulting in an “infodemic,” due to quick and widespread dissemination of both accurate and inaccurate information (3, 4). During the pandemic, social media platforms (i.e., Facebook, Twitter and TikTok) became significant sources of information sharing and searching. In fact, the use of social media platforms increased by 20–87% globally during the pandemic. A recent systematic review revealed that online social media platforms are vulnerable to the spread of incorrect information (5), as the vast amount of health information disseminated on these platforms lacks comprehensive verification (6). A study also revealed that a majority of adults had been exposed to COVID-19-related misinformation, either through social media or instant messaging platforms (7). Such an infodemic has highlighted a population's low level of health literacy as an underappreciated global public health issue (8). However, social media platforms are also central resources of reliable health information and real-time updates (2), as during the pandemic, the public were more dependent on digital resources due to social distancing policies that restricted personal activities and social gatherings. Thus, health literacy is crucial in this era, as this infectious disease crisis arrived at a time of information excess, and integrating all of the available information to make personal health behavioral choices may be challenging for many individuals (9). Moreover, scholars have used a context-specific approach to define the concept of critical health literacy in a pandemic (CHL-P). They define it as the skills required in a pandemic to recognize and effectively address the urgency of action at all levels, the complexity of causes and effects and the evolution of the scientific base. It has also been proposed that CHL-P encourages people to grow in their capacity to critically evaluate and reflect on the contextual requirements for effective behavior (10).

### Health literacy and its consequences

Adequate health literacy is required to deal with the overwhelming amount of inaccurate health information. As defined by Sørensen et al. (11), health literacy refers to “knowledge, motivation, and competencies to access, understand, appraise, and apply health information in order to make judgments and take decisions in day-to-day life concerning healthcare, disease prevention, and health promotion to maintain or improve quality of life over the lifetime”. The World Health Organization defines health literacy as the cognitive and social abilities that affect an individual's motivation and capacity to obtain, absorb and use knowledge to maintain and develop their health (12). Health literacy empowers a person to be proactive in maintaining their health and confers the ability to take action and make well-informed decisions. Limited health literacy has been found to be significantly associated with poor health status, high use of healthcare services, low socioeconomic status, lower education and older age (13). Studies have shown that limited health literacy is indirectly and directly associated with poor health and clinical outcomes (14–16). Data from different developed countries have demonstrated that limited health literacy is associated with the declining use of public health services and information and disease prevention services and poorer self-management of diseases (17). Conversely, individuals with a higher level of health literacy have less frequent use of hospital services and better health status, due to greater disease knowledge, healthier behaviors, greater use of preventative treatment and greater drug compliance (18). Recent research has also demonstrated that health literacy may be seen as a social vaccine, as it enables individuals and communities to reduce the spread of viruses by comprehending and using the information supplied by governments and health authorities (19). Thus, adequate health literacy is essential to manage the current pandemic. Despite the concerns about and importance of COVID-19-specific health literacy, there is little empirical research on this topic, with only one study performed in Taiwan (20) and one in Germany (21). Thus, COVID-19-specific health literacy is a crucial research topic during this global health emergency.

### Health literacy and COVID-19 vaccine hesitancy

In a recent study of COVID-19 vaccine hesitancy conducted in seven European countries, 18.9% of the sample of more than 7,600 respondents were uncertain of their intention to receive the COVID-19 vaccine, and 7.2% reported their unwillingness to be vaccinated (22). Studies have developed models, such as the 5C model (23), to integrate data regarding vaccination behavior. However, COVID-19 vaccines differ from previous vaccines in many aspects, such as the speed of their development, vaccine effectiveness and potential side effects (24). COVID-19 vaccine hesitancy has been shown to be dependent on context, place, time and the type of vaccine (25). A recent study of school principals in Taiwan reported that individuals with limited COVID-19-specific health literacy had a lower intention to be vaccinated against COVID-19 (20). Moreover, a study of Chinese college teachers and students found that the key factors influencing their reluctance to receive the COVID-19 vaccine were beliefs about the safety of the vaccines, attention to and awareness of vaccine-related news and chronic medical issues. However, students' vaccine hesitancy was unrelated to teachers' vaccine hesitancy (26). A recent French study conducted by Montagni et al. (27) also revealed that fake news detection scores of adults were associated with the intention to receive the COVID-19 vaccine. Altogether, these findings highlight that inadequate health literacy may affect individuals' hesitancy to receive the COVID-19 vaccine, as they may be concerned about its safety and affected by news about the vaccine. Misinformation may deeply influence their decision on whether to receive the vaccine. Thus, the association between inadequate health literacy and COVID-19 vaccine hesitancy is worth investigating.

### Importance of health literacy in school personnel

Education is one of the social factors affecting the health literacy of the general population, especially children and adolescents, as they are at their most receptive period of life (28). The long-term development of children's and adolescents' health and well-being is expected to benefit from investments in health literacy (29, 30). Investing in health literacy system capabilities requires a systematic effort and transformation that may multiply and sustain over time and that is resilient to external trends and events, rather than relying solely on organizational and individual behavioral changes (31). Moreover, scholars have reported that improving health literacy across populations and systems is critical to achieving health equity (31). Consequently, it is important to recognize that health literacy in schools is a major public health issue (32). In this regard, schools have long been seen as a crucial setting in which to promote disease prevention and health promotion and develop the health literacy of students, teachers and administrators (33–36). The health literacy of children and adolescents has long been a popular research topic, but equal attention should be given to the health literacy of teachers, as they are responsible for enhancing the students' understanding of health-related topics. Peterson et al. (2001) defined teachers' health literacy as “the capacity of teachers to obtain, interpret and understand basic health information and services, with the competence to use such information and services in ways that enhance the learning of health concepts and skills by school students” (37). Teachers' health literacy may be as important as students' health literacy, as teachers are like health information providers, while students are the consumers (37). However, the prevalence of limited health literacy is concerning. A study of secondary school teachers in Sri Lanka and Iran found that the prevalence of problematic or inadequate health literacy was 31.5 and 50.6%, respectively (38, 39). In Turkey, a study of 500 teachers found that more than 70% of them had extremely low or low levels of health literacy (40). A survey of 1,000 educators conducted in Japan during the COVID-19 pandemic, using the European Health Literacy Survey Questionnaire (HLS-EU-Q47), revealed that educators had gained sufficient health literacy, with a score of 33.5 out of 50 (41). Under the context of COVID-19, a recent study of school principals in Germany revealed a prevalence of 29.3% for limited COVID-19-specific health literacy. These findings demonstrate that sufficient health literacy levels are place- and time-specific. Therefore, research on COVID-19-specific health literacy among school teachers in Hong Kong is urgently needed, especially as research on this topic in Hong Kong has been limited for many years.

### Health literacy in the context of work behavior and stress symptoms

Making appropriate health decisions based on relevant information may be beneficial in occupational and health settings (42). Studies have also shown that limited health literacy may be one of the barriers to the understanding and effectiveness of occupational training (43). A study of young adult workers also revealed that six components of their proposed health literacy structural model, namely, “self-perception,” “a proactive approach to health,” “self-control,” “self-regulation” and “communication and cooperation,” were associated with work ability (44). Although an Iranian study reported no significant relationship between job stress and health literacy (45), other studies have suggested that uncertainty related to COVID-19 has the potential to increase people's stress, anxiety, risk of burnout, fear and frustration levels (46–53). Educators have been accumulating negative psychological symptoms, such as stress, anxiety and depression, due to the closure of educational facilities globally and the need to adapt to new teaching methods since the beginning of the COVID-19 pandemic (54–56). A recent systematic review showed that the estimated prevalence of stress among teachers globally was 30% (57). In the context of Hong Kong, a local survey revealed that more than 85% of teachers felt stressed or over-stressed, and more than one third of people surveyed worked 61 hours or more per week (58, 59). These findings indicate that educators in Hong Kong are an occupational group experiencing a high level of stress since the beginning of the COVID-19 pandemic. In the context of a heavier workload and a greater need for self-management, researchers have hypothesized that self-endangering behavior, including work intensification, extensification and quality reduction, may be used as coping mechanisms (60). However, engaging in these behaviors may be harmful to one's physical health and long-term capacity for employment (60). Burnout syndrome, which includes psychosomatic complaints and exhaustion, and mild to severe mental disorders, such as anxiety and depression, may occur due to work overload or stress (61, 62). Therefore, investigating health literacy and its relationship to self-endangering work behavior and burnout syndrome may facilitate the development of interventions aimed at improving the mental health of teachers globally.

In Hong Kong, the health literacy level of the population is concerning. A recent study reported that 20.7% and 35.2% of the general population had inadequate or problematic health literacy levels, respectively (63). Inadequate levels of health literacy were also found to be prevalent among older adults in Hong Kong (64). However, the health literacy of teachers in Hong Kong has rarely been discussed, even though the unexpected digital revolution in teaching due to the COVID-19 pandemic has brought major teaching challenges, such as technical and motivational problems (65). According to a survey of 1,200 teachers in Hong Kong, conducted by the Hong Kong Federation of Education Workers in November 2020, 85% of teachers felt “relatively high” or “very high” levels of pressure at work during the pandemic. This prevalence is 5% higher than the prevalence reported in the previous year (66).

In this study, we aimed to examine COVID-19-related health literacy in teachers in Hong Kong and explore how different demographic variables are associated with health literacy and how health literacy levels predict the level of COVID-19-related information received, self-endangering work behavior and burnout symptoms. Moreover, we explored how COVID-19-related health literacy affected teachers' decisions regarding and attitudes toward vaccination.

## Methods

This cross-sectional quantitative study was carried out with teachers in primary, secondary and special schools in Hong Kong from April 2021 to February 2022, which was during a period of strict social distancing measures. This study was designed within the framework of the COVID-HL Network, a global research network on health literacy related to COVID-19 that comprises more than 150 researchers from 70 countries (https://covid-hl.eu, accessed on 30 September 2022). The theoretical framework of the present study was health literacy and the infodemic and the survey was validated and adapted from Dadaczynski et al. (67). The survey was translated from English into traditional Chinese based on the conceptual, cultural and linguistic settings in Hong Kong. The translated version was reviewed and rephrased based on feedback from a pilot study of eight participants and the opinions of the authors.

### Participant recruitment

The sample was composed of teachers in primary, secondary and special schools in Hong Kong. Participants were recruited through email invitations. English and Chinese versions of an online survey on the Qualtrics platform were sent to the principals of 1,130 schools, including 561 primary schools, 477 secondary schools and 36 special schools, registered with the Education Bureau in Hong Kong. The school principals were contacted *via* our established school network, with priori verbal agreement granted over the phone. Hardcopies of the surveys were mailed to 243 schools. In addition, we sent invitations *via* our personal and professional networks through social media platforms (e.g., WhatsApp), and respondents were asked to invite eligible participants to participate. Participation was anonymous and voluntary and no incentives were provided. Confidentiality of information was guaranteed. The study protocol was approved by the research ethics committee of Hong Kong Baptist University (REC/20-21/0465).

## Instruments

### Demographic characteristics

Demographic characteristics, including sex, age (years) and school type (primary school, secondary school or special school), were ascertained.

### COVID-19-related health literacy

The 22-item HLS-COVID-Q22 scale (21) was used to measure the participants' capacity to understand and utilize COVID-19-related health information. The validated scale has high internal consistency (α = 0.940) and has been shown to be reliable for measuring COVID-19-related health literacy (21). The participants scored on a 4-point scale, from 1 (very difficult) to 4 (very easy), their perceived difficulties in accessing, understanding, appraising and applying health-related information in the context of the COVID-19 pandemic. Responses for the items were averaged to form the scale score, and the attainment of sufficient health literacy was indicated by an average score of 3. An average score less than or equal to 2.5 indicated inadequate health literacy, while an average score >2.5 but < 3 indicated problematic health literacy (21). A Cronbach's alpha value of 0.957 was obtained in the present study.

### Health information in the context of the COVID-19 pandemic: Attitudes toward vaccination

The adequacy and clarity of health information in the context of the COVID-19 pandemic were measured using the following two items: “On a scale of 1 (very informed) to 5 (insufficiently informed), how well-informed do you feel about the coronavirus or the COVID-19 pandemic?” and “On a scale of 1 (not at all confused) to 4 (very confused), do you feel confused about COVID-19-related information?” (21). The willingness to get vaccinated against COVID-19, if offered, was also measured on a 6-point scale, from “yes, certainly” to “certainly not”, or “I am already vaccinated”. Attitudes toward vaccination were assessed using five self-reported items. Examples of these items included “vaccinations are important to protect me and my family” and “vaccination is compatible with my attitudes”. The participants were asked to rate their agreement of each item using a 4-point scale, from 1 (totally agree) to 4 (do not agree at all). The responses for the items were averaged to calculate the scale score.

### Health promotion and disease prevention in schools

Health promotion and disease prevention in schools were measured using a 15-item questionnaire based on a study by Dadaczynski and Hering (67). The questionnaire measured a range of health issues addressed by schools in the context of the COVID-19 pandemic. The participants rated how they agree with the 15 statements (e.g., “students learned how to eat healthily despite the restrictions due to the coronavirus”), on a 4-point scale, from 1 (not true at all) to 4 (totally true). The responses for each item were summed and averaged to calculate the scale score. Higher scores indicated greater disease prevention and health promotion efforts at the school.

### Exhaustion related to work and psychological discomfort

The three-item “exhaustion” subscale of the Burnout Assessment Tool was used to assess exhaustion related to work, while psychological discomfort was measured using the 5-item “psychosomatic complaints” subscale (68). The participants rated how often the statement applied to them on a five frequency-based response scale, from 1 (never) to 5 (always). The responses for each item were summed and averaged to calculate the total score. The Cronbach's alpha values of the exhaustion and exhaustion subscales in the present study were 0.932 and 0.801, respectively.

### Self-endangering work behavior

Self-endangering work behaviors were assessed using three subscales of self-reported self-endangering work behavior scales (60), including “extensification of work” (six items), “intensification of work” (three items) and “work quality reduction” (three items). The reliability of the scale has been shown previously (60). The participants rated how often the statements applied to them in the past 3 months, on a 5-point scale from 1 (never) to 5 (very often).

### Work-related information

The number of teaching hours per week, the number of working hours per week, the change in the number of working hours per week due to the COVID-19 pandemic and the number of students at the school were collected. The participation status in health-promoting programmes at the participants' schools was also determined (never, < 1 year, 1 to < 2 years, 2 to < 3 years, 3 years or above).

### Perceived general health, presence of any chronic disease and impairment due to health problems

The general health of the respondents was assessed through the self-reported question “How is your overall health?”, on a 5-point scale, from 1 (very good) to 5 (very poor). The participants were asked if they had any chronic or long-term health problems, answering 1 for “no” and 2 for “yes”, and to rate the degree to which their chronic disease impaired their activities of daily living, on a 3-point scale from 1 (no impairment at all) to 3 (severe impairment) (69).

### Statistical analysis

Continuous variables were reported using the mean (M) and standard deviation (SD). Categorical variables were computed as the number (n) and percentage. Mean differences in COVID-19-specific health literacy among demographic groups (i.e., by age, sex, type of schools and number of students) were explored using independent Student's *t-*tests. Potential predictors of COVID-19-specific health literacy levels (i.e., age, sex and level of health promotion and disease prevention in schools) were explored using adjusted multiple linear regression models. Separate adjusted multiple linear regression models were computed to detect the predictive power of COVID-19-specific health literacy for COVID-19 vaccine hesitancy. Adjusted multiple linear regression models were used to explore how COVID-19-specific health literacy affected one's perceived general health. Mean score differences in self-endangering work behavior (i.e., extensification of work, intensification of work and work quality reduction), burnout symptoms (i.e., exhaustion related to work situation and psychosomatic complaints) and the level of knowledge of COVID-19- or pandemic-related information were compared between health literacy groups using an independent samples Student's *t-*test or one-way analysis of variance (ANOVA). All data analyses were performed using SPSS 27.0 for Windows (IBM, Armonk, NY, USA). Statistical significance was defined as a *p*-value < 0.05.

## Results

### Sample characteristics

Of the 366 teacher participants (Table 1), 46.4% were male, and the average age was 38.3 (SD = 9.72) years. Almost half of the participants (45.1%) worked in secondary schools, 33.8% in primary schools and 21.2% in special schools. The average number of teaching hours per week was 21.9 (SD = 10.15), and the average number of working hours per week was 45.4 (SD = 16.18, ranging from 4 to 115 h). The number of working hours per week was higher during than before the COVID-19 pandemic for 51.2% of the participants, about the same for 36.0% of the participants and lower during than before the COVID-19 pandemic for 12.7% of the participants. The average number of students at the school was 516.16 (SD = 315.9).

**Table 1:** HLS-COVID by participants characteristics using *t-*tests and ANOVA (*N* = 366).

**Variables**		***N* (%)**	**HLS-COVID**	
			**Mean (SD)**	***p*-value**
Gender	Male	169 (46.6)	2.97 ± 0.41	0.727
	Female	195 (54.4)	2.98 ± 0.43	
Type of School	Primary schools	123 (33.8)	3.06 ± 0.46	< 0.001**
	Secondary schools	164 (45.1)	3.01 ± 0.39	
	Special schools	77 (21.2)	2.77 ± 0.34	
Weekly working hours change	Lower than before the COVID-19 pandemic	46 (12.7)	3.03 ± 0.39	< 0.001**
	About the same	130 (36.0)	3.09 ± 0.42	
	Higher than before the COVID-19 pandemic	185 (51.2)	2.88 ± 0.41	
Number of students at school	≤ 600	206 (57.9)	2.93 ± 0.39	0.009*
	600 or above	150 (42.1)	3.05 ± 0.46	
Level of informing on COVID-19 related information	Well or very well informed	105 (28.8)	3.15 ± 0.45	< 0.001**
	Poor/satisfactory	260 (71.2)	2.91 ± 0.39	
Level of confusion due to COVID-19 related information	Not at all*/*a little confused	279 (77.3)	3.04 ± 0.39	< 0.001**
	Quite confused*/*very confused	82 (22.7)	2.76 ± 0.45	
		**Number**	**Mean (SD)**	
Age		335	38.32 ± 9.72	
Number of students at school		349	516.16 ± 315.9	
Weekly working hours		358	45.4 ± 16.18	
Weekly teaching hours		356	21.9 ± 10.15	

SD, standard deviation.

*p < 0.05, **p < 0.01.

### Differences COVID-19-related health literacy according to participants' characteristics

There was no significant sex difference in HLS-COVID scores (males: *M* = 2.97, SD = 0.41; females: *M* = 2.98, SD = 0.43). A one-way ANOVA revealed that there was a statistically significant difference in HLS-COVID scores between participants who worked in primary schools, secondary schools and special schools (*F*_[2, 351]_ = 12.59, *p* < 0.001). Tukey's honestly significant difference (HSD) test for multiple comparisons showed that participants who worked in special schools had a significantly lower HLS-COVID score than those who worked in primary schools (*p* < 0.001, 95% confidence interval [CI] = −0.1699, −0.4036) or secondary schools (p < 0.001, 95% CI = −0.3467, −0.1235). There was no statistically significant difference in HLS-COVID scores between participants working in primary schools and those working in secondary schools (*p* = 0.296).

A one-way ANOVA revealed a statistically significant difference in HLS-COVID scores according to the change in the number of working hours per week during the pandemic (*F*_[2, 349]_ = 9.667, *p* < 0.001). Tukey's HSD test for multiple comparisons found that participants with a higher number of working hours per week during the COVID-19 pandemic had significantly lower HLS-COVID scores than individuals with a lower number of working hours per week during the COVID-19 pandemic (*p* = 0.03, 95% CI = −0.2797, −0.0115) and individuals with no change in the number of working hours per week (*p* < 0.001, 95% CI = −0.3003,−1.1117). An independent samples Student's *t-*test revealed a statistically significant difference in HLS-COVID scores between the group of participants who had 600 or fewer students at their schools and the group of participants who had more than 600 students at their schools (*t*_[354]_ = 2.274, *p* = 0.009). The results also showed that the participants who felt well informed or very well informed about COVID-19-related information had significantly higher health literacy scores (*M* = 3.15, SD = 0.45) than those who felt insufficiently or poorly informed about COVID-19-related information (*M* = 2.91, SD = 0.39; *t*_[350]_ = 5.38, *p* < 0.001). The participants who felt not at all or a little confused about COVID-19-related information (*M* = 3.04, SD = 0.39) had higher health literacy scores than those who felt very confused.

Table 2 provides the descriptive statistics of health-related variables. The average total HLS-COVID-Q22 score for the total sample was 2.98 ± 0.42. Sufficient health literacy was observed in 50.8% of the participants, whilst 47.4% participants had insufficient health literacy (including problematic or inadequate literacy levels). The majority of the participants (71.3%) had insufficient, poor or satisfactory pandemic-related information, and 77.3% of the participants felt not at all or a little confused about pandemic-related information. Almost half of the participants (49.0%) had already received a COVID-19 vaccine. The total mean scores for attitudes toward vaccination, health promotion and disease prevention in schools and perceived general health were 2.13 (SD = 0.65), 44.6 (SD = 6.49) and 2.65 (SD = 0.80), respectively. Most of the participants (82.7%) did not present with any chronic disease or long-lasting health problems. The majority of the participants (72.4%) were not at all impaired by health problems.

**Table 2:** Descriptive table of health-related variables in the sample (*N* = 366).

**Variables**		***n* (%)**
HLS-COVID-Q22	Sufficient health literacy	186 (50.8)	
	Problematic health literacy	130 (36.5)	
	Inadequate health literacy	40 (10.9)	
Level of informing on COVID-19 or pandemic related information	Well or very well informed	105 (28.8)	
	Insufficient*/*poor	260 (71.3)	
Level of confusion due to COVID-19 related information	Not at all*/*a little confused	279 (77.3)	
	Quite confused*/*very confused	82 (22.7)	
Coronavirus vaccination readiness	Certainly*/*Likely	119 (32.6)	
	Maybe	37 (10.1)	
	Certainly not*/*Unlikely	30 (8.2)	
	Already vaccinated	179 (49.0)	
Presence of any chronic disease or long-lasting health problem	No	301 (82.7)
	Yes	63 (17.3)	
Impairment by health problems	Not at all impaired	260 (72.4)
	Moderately impaired	93 (25.9)	
	Strongly impaired	6 (1.7)	
		**Number**	**Mean (SD)**
Covid-19-related health literacy (HLS-COVID-Q22)		356	2.98 ± 0.42
Attitudes about vaccination		358	2.13 ± 0.65
Health promotion and prevention in schools		355	44.6 ± 6.49
Perceived general health		364	2.65 ± 0.80

SD, standard deviation.

Table 3 shows the descriptive statistics for work satisfaction, self-endangering work behavior (i.e., extensification of work, intensification of work and work quality reduction) and secondary burnout symptoms (i.e., exhaustion related to work situations and psychosomatic complaints) for the entire sample and the differences between the two health literacy groups: sufficient health literacy (*n* = 186) and insufficient health literacy (*n* = 170). Respondents with sufficient health literacy (*M* = 2.95, SD = 0.87) had significantly lower intensification of work scores than those with insufficient health literacy (*M* = 3.23, SD = 0.88; *t*_[352]_ = 3.004, *p* = 0.003). After excluding participants who had already been vaccinated, those who had sufficient health literacy (*M* = 1.92, SD = 1.09) scored lower for their attitudes toward COVID-19 vaccination (i.e., felt more positive about COVID-19 vaccination) than those with insufficient health literacy (*t*_[180]_ = 4.168, *p* < 0.001). In addition, teachers with sufficient health literacy (*M* = 2.60, SD = 0.72) felt more informed about COVID-19-related information than those with insufficient health literacy (*M* = 2.97, SD = 0.62; *t*_[354]_ = 5.142, *p* < 0.001). Moreover, those with sufficient health literacy (*M* = 1.91, SD = 0.67) felt less confused about COVID-19-related information than those with insufficient health literacy (*M* = 2.24, SD = 0.63; *t*_[350]_ = 4.68, *p* < 0.001).

**Table 3:** Work satisfaction, self-endangering work behavior and secondary burnout symptoms by health literacy level using *t-*tests (*N* = 366).

**Health literacy level**	**Mean (SD)**	**Mean diff**.	***p***	**Mean (SD)**	**Mean diff**.	**p**	**Mean (SD)**	**Mean diff**.	***p***
	**Work satisfaction**			**Exhaustion related to work situation**			**Psychosomatic complaints**		
**Sufficient**	3.05 (0.81)	−0.067	0.433	3.33 (0.94)	0.033	0.353	2.52 (0.78)	0.466	0.546
**Insufficient**	2.98 (0.77)			3.36 (0.88)			2.57 (0.66)		
	**Extensification of work**			**Intensification of work**			**Quality reduction**		
**Sufficient**	3.31 (0.80)	0.079	0.333	2.95 (0.87)	0.279	**0.003**	2.26 (1.02)	−0.027	**0.007**
**Insufficient**	3.39 (0.72)			3.23 (0.88)			2.53 (0.85)		
	**Attitude about COVID-19 Vaccination**			**Level of informing of COVID-19 related information**			**Level of confusion due to COVID-19 related information**		
**Sufficient**	1.92 (1.09)	0.705	**< 0.001**	2.60 (0.72)	0.368	**< 0.001**	1.91(0.67)	0.362	**< 0.001**
**Insufficient**	2.62 (1.19)			2.97 (0.62)			2.24 (0.63)		

Bold figures indicates p < 0.05.

SD, standard deviation.

Multiple linear regression analyses were performed to explore the association between health literacy level and other potential influential factors (i.e., age, sex and number of working hours and teaching hours per week). Table 4 provides the regression coefficients for predicting the health literacy level. The model was adjusted for age and sex. A significant regression equation was found (F_[4, 312]_ = 6.500, *p* < 0.001) with an R-squared value of 0.077. The results showed that age and the number of teaching hours per week were significant predictors of HLS-COVID scores. Specifically, age positively predicted the HLS-COVID score, and the number of teaching hours per week negatively predicted the HLS-COVID score. Separate multilinear regression models adjusted for age, sex and the presence of chronic illness were generated to predict attitudes toward vaccination (Table 5). The level of COVID-19-specific health literacy was negatively associated with attitudes toward COVID-19 vaccination, while the level of confusion about COVID-19-related information was positively associated with attitudes toward COVID-19 vaccination (i.e., less agreeable to vaccination; F_[6, 158]_ = 5.865, *p* < 0.001), with an R-squared value of 0.182.

**Table 4:** Regression model of predicting health literacy by age, weekly working hours and teaching hours.

**Variables**	**B(95%CI)**	**β**	***p***
Gender	0.02 (−0.089, 0.093)	0.002	0.952
Age	0.001 (0.000,0.003)	0.140	**0.011**
Weekly working hours	0.003 (0.000, 0.006)	0.106	0.054
Weekly teaching hours	−0.009 (−0.013, −0.004)	−0.206	**< 0.001**

Bold figures indicates *p* < 0.05.

**Table 5:** Regression model of predicting attitude about vaccination by age, sex, level of informing of COVID-19 related information and level of confusion due to COVID-19 related information.

**Variables**	**B(95%CI)**	**β**	***p***
Gender	0.68 (−0.263, 0.399)	0.030	0.684
Age	−0.02 (−0.005, 0.001)	−0.086	0.249
Covid-19-related health literacy	−0.817 (-1.251, −0.382)	−0.311	**< 0.001**
Level of informing on COVID-19 related information	−0.046 (−0.013, −0.004)	−0.026	0.749
Level of confusion due to COVID-19 related information	0.332 (0.035, 0.628)	0.177	**0.029**
Presence of chronic illness or long-term health problem	−0.106 (−0.320, 0.109)	−0.071	0.333

Bold figures indicates *p* < 0.05.

## Discussion

The present study aimed to examine whether different demographic variables, workplace stress levels, levels of COVID-related information received and burnout-related behaviors were associated with health literacy in teachers in Hong Kong. This is one of the first studies to explore COVID-19-related health literacy and its associated factors in teachers in Hong Kong. We found that approximately half (50.8%) of the participants had sufficient health literacy, while 47.4% had “problematic health literacy” (36.5%) or “inadequate health literacy” (10.9%). The prevalence of inadequate health literacy (10.9%) in our study group was markedly lower than the prevalence previously reported in other groups (e.g., 50.9% in older adults) in Hong Kong and also lower than the reported in non-healthcare settings (46.9%) in Malaysia (70). In a recent study in Germany that focused on HLS-COVID scores, the prevalence of low health literacy (i.e., problematic or inadequate health literacy) in the general population ranged from 50.4% in the first wave of the COVID-19 pandemic (April 2020) to 38.2% in the third wave (December 2020) (71). The prevalence of limited health literacy was markedly lower in our study group than the prevalence reported in a sample of 1,360 participants in Shanghai, China (10.9% vs. 85%) (72). The mean HLS-COVID score observed in our study (2.98 ± 0.42) indicates that the participants attained sufficient health literacy; however, it was slightly lower than the average score reported for school principals in Taiwan (*M* = 3.2, SD = 0.4) (20). These findings demonstrate that the prevalence of low health literacy varies across different countries and regions, and the present study contributes part of the picture of COVID-19-specific health literacy among school personnel in Hong Kong. Even in this group of well-educated individuals, nearly half of them had a low level of health literacy. Therefore, interventions focusing on increasing health-related knowledge are urgently needed to improve health literacy among school teachers.

We found no significant difference in health literacy according to sex. This is in line with a study in Hong Kong which revealed no significant sex disparities in any aspect of health literacy (70). However, other studies have shown that females tend to have a higher level of health literacy than males (73, 74). A recent study in Taiwan also reported that female school principals tended to have a higher level of COVID-19-related health literacy (20). A study in Korea revealed that female adults tended to have a higher level of health literacy in the domains of learning about medical paperwork, directions on medication bottles and written information from their healthcare professional (75). Whilst these findings demonstrate that the effect of sex on health literacy remains varied, the present study findings suggest that individuals with similar occupations do not have a sex difference in health literacy.

Significant differences were observed in health literacy between participants working in different types of school. Those working in special schools had a lower level of COVID-19-related health literacy than those working in either primary or secondary schools, but there was no statistically significant difference between participants who worked in primary schools and those who worked in secondary schools. There is no existing evidence for a lower level of health literacy among teachers working in special schools. However, a cross-sectional study of primary and secondary school teachers in Çorum, Turkey (*n* = 580) conducted in 2015 using the Newest Vital Sign scale revealed that there was no significant difference in the health literacy level between teachers working in primary and secondary schools (40). As there are few studies showing that working in different types of school affects health literacy, the significant difference detected in the current study may be attributable to the small sample of participants who worked in special schools. Further studies may be needed to validate this difference in health literacy according to school type.

Significant differences in health literacy were observed between participants who had a higher number of working hours per week during than before the pandemic and those with a lower or about the same number of working hours per week. However, a study of Filipino domestic workers revealed that the number of working hours per week was not associated with the health literacy level (76). Meanwhile, another study of 500 young Japanese nurses and care workers showed that working at their own pace, maintaining a work–life balance, regularly performing self-check-ups and attending lectures and workshops were factors that were significantly associated with a high health literacy level (77). This may explain our findings, as an increase in the number of working hours per week during the pandemic may have affected the working pace and work–life balance of the teachers. Thus, teachers may have had less time to access, understand and appraise vast amounts of health information during the COVID-19 pandemic.

In the present study, teachers with sufficient health literacy felt significantly more well-informed and significantly less confused about COVID-19-related information than those with insufficient health literacy. This finding was consistent with the finding of a study of HLS-COVID scores in the general population in Germany (21). As the perceived ease or difficulty in appraising health information is one of the core dimensions of health literacy, feeling less confused or better informed about health information may indicate a greater ability to appraise COVID-19-related information (11). A study also reported that a low level of health literacy is the underlying cause of confusion for many people when seeking health-related information (78). Consistently, the present study revealed that teachers with insufficient COVID-19-specific health literacy were more likely than those with sufficient literacy to be less informed and more confused about COVID-19-related information, which is similar to the situation for individuals with insufficient general health literacy. We found that age and the number of teaching hours per week were significant predictors of COVID-19-specific health literacy, with older age being associated with a higher level of literacy, and a higher number of working hours per week being associated with a lower level of literacy. Several studies in Europe, Taiwan and Australia have suggested that a young age is associated with limited health literacy in the general population (13, 79–82). In contrast, other studies have reported that old age is significantly associated with limited health literacy (40, 83). A health literacy study of community-dwelling individuals in Hong Kong reported that age was negatively correlated with the health literacy level (63). Further studies are needed to elucidate the association between age and health literacy, especially in the context of COVID-19.

We found that a higher number of working hours per week was associated with a lower level of COVID-19-specific health literacy. Although studies have shown that some of the dimensions of the constructed health literacy model, such as “self-regulation” and “self-perception,” were associated with one's work ability (84), the relationship between workload, the number of working hours and health literacy has rarely been discussed. Assessing, understanding and appraising is a time-consuming process, and teachers may not have had time to select or distinguish accurate health information under the heavy workload they experienced during the pandemic (85). Additionally, our study revealed that teachers with insufficient health literacy had significantly higher frequencies of two types of self-endangering behaviors—“intensification of work” and “work quality reduction”—than those who had sufficient health literacy. As mentioned, a previous study demonstrated that health literacy may be associated with work abilities (84). However, research exploring the relationship between health literacy and self-endangering work behavior is lacking. Studies have proposed that self-endangering work behavior is a form of coping in response to work overload (60). Further studies may be needed to explore whether teachers with limited health literacy are more likely to experience work overload and perform self-endangering work behavior.

Teachers who had sufficient health literacy had a significantly lower score for their attitudes toward vaccination (i.e., had a more positive attitude) than teachers with insufficient health literacy. Similarly, our regression model demonstrated that a more positive attitude toward vaccination was associated with a higher level of COVID-19-related health literacy. This finding is consistent with the finding of a COVID-19-related health literacy study of Taiwanese school principals, in which principals with higher COVID-19-related health literacy reported a lower level of vaccine hesitancy (20). However, a previous systematic review also concluded that the relationship between health literacy and vaccine intention remains unclear, as COVID-19 vaccine hesitancy or acceptance was found to be affected by factors including the country, age and type of vaccine (86). Thus, improving teachers' COVID-19-related health literacy may also be a strategic approach to improve teachers' individual vaccine acceptance. However, vaccine acceptance may also be influenced by other factors, such as age and the type of vaccine. Thus, further studies are needed to facilitate our understanding of the relationship between health literacy and vaccine acceptance.

## Study limitations

This study has some limitations. First, we used a purposeful convenience sample, which precludes the generalisability of the findings, as they may not be typical of all school teachers. Second, the cross-sectional nature of the study is a shortcoming, as it only allowed the examination of associations between variables, and not causality. This design also hindered our understanding of changes in the variables over time. Third, the participants' responses were collected on a self-reported basis. The validity of our study findings may be limited due to “social desirability” bias, that is, teachers may have tended to respond with well-accepted social behaviors in the questionnaire. However, it is worthwhile to note that the teacher participants were informed about the anonymity of their responses. Fourth, to increase the response rate, the sampling duration was extended to 11 months, which covered the third and fourth waves of the COVID-19 pandemic in Hong Kong. During this time, Hong Kong transitioned between half-day face-to-face lessons at schools and virtual learning at home. The change to online learning and teaching may have led to an underestimation of the changes in workload during the COVID-19 pandemic. Furthermore, most of the data were gathered when the Hong Kong immunization programme was in its early stages and vaccination pass procedures had not yet been implemented. As a result, the attitudes and readiness toward vaccination may not be applicable to the current situation in Hong Kong. Future studies should validate the findings by using a larger, more representative sample.

## Conclusions

The present study contributes to our understanding of health literacy among school teachers in Hong Kong. Our study findings revealed the prevalence of limited health literacy among school teachers in Hong Kong, as almost half of the teachers had “problematic health literacy” or “inadequate health literacy”. Data from the present study also indicate that a higher level of COVID-19-related health literacy among teachers is associated greater knowledge of pandemic-related information, less confusion about COVID-19-related information, a more positive attitude toward vaccination, a higher level of health promotion and disease prevention in schools, a lower level of psychosomatic complaints and better perceived general health.

## Data availability statement

The raw data supporting the conclusions of this article will be made available by the authors, without undue reservation.

## Ethics statement

The studies involving human participants were reviewed and approved by Research Ethics Committee of Hong Kong Baptist University (REC/20-21/0465). The participants provided their written informed consent to participate in this study.

## Author contributions

Conceptualization and study design: SL, PA, AL, KD, and OO. Coordination of the study: SL, JM, and EC. Funding requisition: SL. Data collection: SL, JM, EC, PA, and AL. Data analysis and writing up of the manuscript: SL and ES. Review of the manuscript: SL, ES, JM, EC, PA, KD, and OO. All authors have read and agreed to the published version of the manuscript.
